# Relationship of microbial communities and suppressiveness of *Trichoderma* fortified composts for pepper seedlings infected by *Phytophthora nicotianae*

**DOI:** 10.1371/journal.pone.0174069

**Published:** 2017-03-27

**Authors:** Margarita Ros, Iulia Raut, Ana Belén Santisima-Trinidad, Jose Antonio Pascual

**Affiliations:** 1 Department of Soil and Water Conservation and Organic Waste Management, Centro de Edafología y Biología Aplicada del Segura, CSIC, Murcia, Spain; 2 National institute for Research & Development in Chemistry & Petrochemistry – ICECHIM, Biotechnology & Bioanalysis group, Bucharest, Romania; Dong-A University, REPUBLIC OF KOREA

## Abstract

The understanding of the dynamic of soil-borne diseases is related to the microbial composition of the rhizosphere which is the key to progress in the field of biological control. *Trichoderma* spp. is commonly used as a biological control agent. The use of next generation sequencing approaches and quantitative PCR are two successful approaches to assess the effect of using compost as substrate fortified with two *Trichoderma* strains (*Trichoderma harzianum* or *Trichoderma asperellum*) on bacterial and fungal communities in pepper rhizosphere infected with *Phytophthora nicotianae*. The results showed changes in the bacterial rhizosphere community not attributed to the *Trichoderma* strain, but to the pathogen infection, while, fungi were not affected by pathogen infection and depended on the type of substrate. The *Trichoderma asperellum* fortified compost was the most effective combination against the pathogen. This could indicate that the effect of fortified composts is greater than compost itself and the biocontrol effect should be attributed to the *Trichoderma* strains rather than the compost microbiota, although some microorganisms could help with the biocontrol effect.

## Introduction

Sweet pepper (*Capsicum annum L*.) is one of the main horticultural crops in Murcia (South-east Spain), with over 1334 ha of total cultivated area under greenhouses. In this area, *Phytophthora nicotianae* (*P*. *nicotianae*) has been reported as the main causal agent of *Phytophthora* root rot in pepper plants [1]. The management of this disease is based on phenylamide fungicides but fungicide-tolerant strains have been detected [2].

Composts can be used as growing media as a partial substitute of peat to produce high quality seedlings, thus increasing the guarantees of crop success after transplanting [3, 4]. Furthermore, composts have been proved to suppress a wide variety of soil-borne plant pathogens at nursery and field conditions e.g. *Phytophthora nicotianae* [5], *Fusarium oxysporum* [6, 4], or *Pythium ultimum* [7].

Some specific *Trichoderma* spp. strains have been widely used as biological control agents (BCAs) and can reduce the severity of plant diseases by inhibiting plant pathogens in the soil/substrate through their highly potent antagonistic and mycoparasitic activity [8]. Moreover, some *Trichoderma* strains can interact directly with roots, increasing plant growth potential, resistance to disease and tolerance to abiotic stress [9]. A combination of antagonistic microbes with high quality compost may be more efficient in inhibiting disease than using single antagonistic microbial strains or compost alone [10, 11].

BCAs should not have any effects on non-target organisms [12]. However, direct interactions between antagonistic strains of *Trichoderma* spp. and other bacteria/fungi do not specifically target plant pathogens but also the saprophytic micro-organisms. Since application of biocontrol strains of *Trichoderma* spp. has the potential to modify the soil/compost microbiota, its impact on non-target micro-organisms has to be studied [13]. Several studies have been conducted to investigate non-target effects of BCAs on soil microbial communities [14, 15] and no studies have been conducted on compost/peat substrates. A key to progress in the field of biological control is focused on the dynamic interactions between the biocontrol agent, the pathogen and rhizosphere [16]. Next generation sequencing (NGS) or quantitative polymerase chain reaction (qPCR) help to study bacteria and fungi community giving new levels of specificity, accuracy, and detection thresholds superior to traditional dilution plating techniques [17, 18].

We hypothesized that combination of *Trichoderma* species and compost can produce fortified substrates to control plant pathogens. Therefore, the objective was to evaluate the effect of two *Trichoderma* species on compost suppresiveness against *P*. *nicotianae* in pepper seedlings. Rhizosphere microbial community (bacterial and fungi) of the different substrates obtained was studied by NGS to determine the influence of *Trichoderma* or pathogen inoculation on the indigenous microbial communities of the substrates. The quantitative PCR (qPCR) technique was also used to determine the dynamic of interaction between the two Trichoderma strains and the *P*. *nicotianae*.

## Materials and methods

### Fungal strains

Both biological control agents (BCAs), *Trichoderma harzianum* T78 (*T*. *harzianum*) (CECT 20714, Spanish Type Culture Collection) (Microgaia Biotech, S.L.) and *Trichoderma asperellum* T36 (*T*. *asperellum*) (ICECHIM institute, Romania) were grown on Potate dextrose agar (PDA, Sharlau, Spain), autoclaved at 121°C for 20 min and amended with 100 mg L^-1^ sterilized streptomycin. The cultures were incubated at 28°C for 7 days. The mycelia were recovered with sterilized distilled water from the surface of Petri dishes using a drigalski spatula.

The pathogen *Phytophthora nicotianae* (*P*. *nicotianae*) was isolated from pepper plants showing disease symptoms (damping off) and it was produced by transferring one agar plug (5 mm) of 7-day-old mycelia on pea agar medium (100 g L^-1^ of ground peas, 100 mg L^-1^ of β-sitosterol and 20 g L^-1^ of technical agar, adjusted to pH 5.5), autoclaved at 121°C for 20 min and amended with 100 mg L^-1^ sterilized streptomycin. The culture was incubated at 28°C for 10 days. The mycelium was recovered from the content of two Petri dishes and mixed with 500 mL sterile distilled water using a blender.

### Suppressiveness bioassay

Mixed compost/peat for substrate (1:1 w/w) was assayed, following good agronomic results shown by Blaya et al. [5, 19]. Composts made from vineyard wastes (Microgaiga, S.L. Murcia, Spain) and black peat (Klasmann) were used for the bioassay and their physico-chemical and chemical characteristics were: Compost: pH 6.71; Electrical conductivity (mS cm^-1^) 1.89; N (%) 0.89; P (%) 0.06; K (%) 0.69; C/N 43.58; Humidity (%) 69.93. Peat: pH 6.34; Electrical conductivity (mS cm^-1^) 0.51; N (%) 0.97; P (%) 0.7; K (%) 0.6; C/N 50; Humidity (%) 70.

Four treatments were assayed, (CPTh) compost and black peat fortified with *T*. *harzianum* (10^6^ cfu g^-1^ substrate); (CPTa) Compost and black peat fortified with *T*. *asperellum* (10^6^ cfu g^-1^ substrate); (CP) Compost and black peat; (P) Black peat (100%). Seeds of pepper (*Capsicum annuum cv*. *Lamuyo*) (surface sterilized in 10% NaClO for 3 min, rinsed three times in sterile water and dried in sterilized paper) were sown in trays of 150 pots, with one seed per pot, with a covering of vermiculite. Germination was carried out in a germination chamber at 28±1°C and 70–75% relative humidity. Once seeds germinated, trays were located in a growth chamber (25±1°C day (16h); 23±1°C night (8h); 70–75% relative humidity). Seedlings were fertilized one day per week (0.495g NO_3_NH_4_ L^-1^; 0.33 g KNO3 L^-1^; 0.1275 g PO_4_H_2_NH_4_ L^-1^) and watering according to their water holding capacity. Eight trays with six pots were used for each treatment. Three trays for control (n = 18) and five trays inoculated with the pathogen (n = 30).

Seedlings with two true leaves (14 days after sowing) were inoculated with the pathogen (*P*. *nicotianae*) (n = 30). The inoculation dose for all treatments was 10^5^ cfu g^-1^ substrate (P_CPTh; P_CPTa; P_CP; P_P). Substrate samples were collected when the experiment was set up and when seedlings were harvested four weeks after sowing (rhizosphere) and stored at -80°C for molecular analysis.

Seedling infection by *P*. *nicotianae* was recorded every day and the cumulative number of infected plants was also recorded from the day after pathogen inoculation. Percentage of dead seedling was calculated as the percentage of diseased plants out of total number of growing plants.

### DNA extraction

Extractions of total DNA from substrates of each treatment inoculated and not inoculated with pathogen at the end of the experiment (0.5 g) were carried out in triplicate. The DNA extraction was carried out using the FastDNA^®^ Spin Kit for soil (Q-Biogene, Carlsbad, CA, USA) following the manufacturer´s instructions. DNA concentrations of samples were determined using a NanoDrop^®^ ND-1000 Spectrophotometer (Thermo Fisher Scientific Inc., USA) and stored at -20°C until required.

### Next Generation Sequence (NGS)

For molecular analysis of bacterial communities, variable regions V1, V3, V6 and V1-V2 of the 16S rRNA gene were amplified using the four primers pairs 8F/120R, F388/R534, F968/R1073 and 8F/R361 [20, 21] and for fungi ITS1 region was amplified with a pair of primers ITS2/ITS5 [22]. Each replicate sample was amplified, and amplicons for the triplicate samples were purified using the kit QIAquick PCR Purification Kit (Qiagen, Hilden, Germany), and composited together into equimolar concentration prior to sequencing. For bacterial PCR amplification, each 25 μl PCR mix contained the following regents: 1X KAPA2G Fast HotStart ReadyMix2 (2X) (Kapa Biosystems, Boston, MA, USA), 1.5 mM MgCl_2_, 0.5 μM of each primer, and 5 μl of DNA. The thermal cycler conditions were firstly 15 cycles of denaturation at 90°C for 30s, amplification with a temperature gradient of 70°C-50°C for 30 s and a final extension of 72°C for 30s. Secondly, samples had 30 cycles of denaturation at 94°C for 45 s, amplification at 50°C for 45 s and a final extension of 72°C for 45 s.

For fungi amplification, each 25 μl PCR mix contained the following regents: 1X PCR buffer (Biotools, Madrid, Spain), 1.5 mM MgCl_2_, 0.4μM of each primer, 0.5 mg mL^-1^ BSA, 200μM dNTPs mix, 2. 5 U TaqPolymerase, and 2 μl of DNA. The thermal cycler conditions were firstly 1 cycle of denaturation at 95°C for 5 min, followed by 35 cycles of denaturation at 95°C for 1 min, amplification at 56°C for 40 s, extension of 72°C for 1 min, and a final extension of 72°C for 7 min.

A library was created using Ion Plus Fragment Library Kit, and barcodes were added by Ion Xpress ^™^ Barcode Adapters 1–96 Kit (Life Technologies, Carlsbad, CA, USA). The template preparation was performed by Ion OneTouch^™^ 2 System and the Ion PGM^™^ Template Kit OT2 400 (Life Technologies, Carlsbad, CA, USA). Finally, the platform sequenced the samples using Ion Torrent PGM (Life Technologies, Carlsbad, CA, USA) with the kit Sequencing Kit Ion PGM 400 in chips Ion 318 Chip kit and Ion 314 Chip kit.

Sequences were analyzed with software package QIIME v1.8.0. [23] and USEARCH v7.0.1090 [24, 25] following the protocol 16s profiling analysis pipeline recommended by Brazilian Microbiome Project (http://brmicrobiome.org).

Briefly, before sequences processing, it was carried out a quality-filtering step with QIIME, where sequences smaller than 60bp or with a mean quality score below 25 were removed. After that, primers and barcodes were removed and a chimera filter (QIIME) was used. De-replication and singletons discarding steps were performed using USEARCH software. The remaining high quality sequences were grouped in operational taxonomic units (OTUs) following open-reference OTU picking protocol for QIIME, where sequences were clustered against the Green Genes v13_8, using the uclust ref algorithm at 97% similarity. Sequences not matching the database were subsequently clustered *novo*. A representative set of OTUs was generated and then the taxonomy of each OTUs was assigned using the same database.

For fungi we used the software packages QIIME v1.8.0. [23], USEARCH v7.0.1090 [24, 25] and ITSx extractor [26] following *ITS Profiling Data Analysis Pipeline* protocol recommend by Brazilian Microbiome Project (http://brmicrobiome.org). As in the previous protocol, during the quality-filtering step, sequences smaller than 60bp or with a mean quality score below 25 were removed. Primers were removed and a chimera filter (QIIME) was used. De-replication step was carried out, and Singletons were eliminated, both with USEARCH software. The variable regions of the remaining sequences were extracted with the ITSx extractor. These sequences were grouped in OTUs with the Usearch with Uparse method. The remaining high quality sequences were grouped in operational taxonomic units (OTUs) following open-reference OTU picking protocol for QIIME, where sequences were clustered against the UNITE database using the uclust ref algorithm at 97% similarity.

### Quantitative PCR (qPCR)

Quantification of *T*. *harzianum*, *T*. *asperellum* and *P*. *nicotianae* were performed in a 7500 Fast Real-time PCR system (Applied Biosystems, Waltham, MA, USA), with Microamp^®^ Fast Optical 96-Well Reaction plate with barcode (Life Technologies, Carlsbad, California). The real-time PCR mixture in a final volume of 25 μl contained a final concentration of 1X Taqman Universal Master mix II (Life Technologies, Carlsbad, California), 200 μM each dNTPs, 0.3 μM each primer (Roche Diagnostics, Germany), 0.1 μM probe, 0.2 mg mL^-1^ BSA and 5 μl of DNA sample.

For *P*. *nicotianae* and *T*. *harzianum* primers and probes were described in Blaya et al. [1] and Lopez—Mondejar et al. [27] respectively. For *T*. *Asperellum*, we designed a new primers and probe on the transcription enhancer factor-1 (TEF-1) DNA binding site: TaspFw: 5’-GGCAGCAACCCCGCTAT-3’; TaspRv: 5’-ACGACGCGATTGAGCAAATA-3’ and Tasp-Pr: 5’-6FAM-CCACTGCACCTCTTCCATCACCCA-ZEN/IBFQ-3’). The efficiency was 95% and sensibility 10 copies. The specificity was shown on S1 Table

The thermocycling conditions for both *Trichoderma* strains and the pathogen *P*. *nicotianae* were 95°C for 10 min, followed by 40 cycles of 95°C for 10 s and 60°C for 40 s. Each sample was analyzed in triplicate and to control the potential presence of PCR inhibitions and internal positive control (IPC) (TaqMan^®^ Exogenous Internal Positive Control Reagents, Applied Biosystems^®^) were included in all reactions according to the manufacturer´s recommendations. Each run contained one negative (bdW) and a DNA positive control.

### Statistical analysis

Results from the suppressive bioassay and quantification of BCAs and *P*. *nicotianae* were subjected to the non-parametric Kruskal-Wallis test and median P-values ≤ 0.05 were considered significant. Statistical analyses were performed using SPSS 23.0 software (SPSS Inc., Chicago, IL, USA). Principal Component Analysis of bacterial and fungal communities was performed with Canoco for Windows 4.5.

## Results

### Suppressive effect and *P*. *nicotianae* abundance in the different substrates

**Fig 1:**
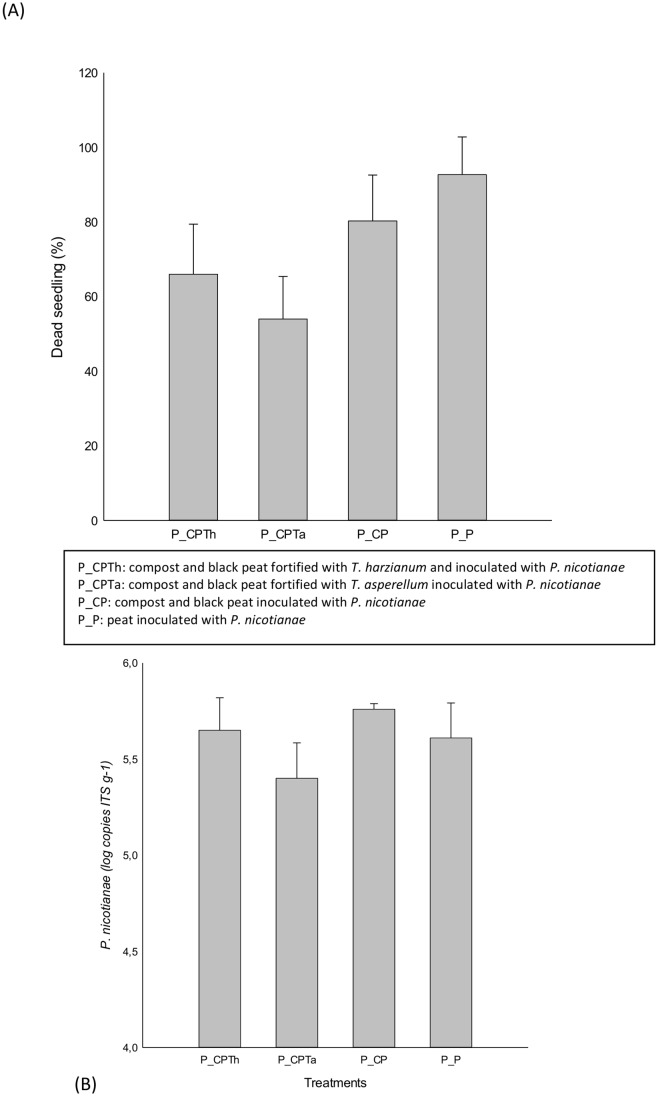
Percentage of dead seedling by *P*. *nicotianae* (A) and quantification of *P*. *nicotianae (B)* for each treatment after harvesting. (P_CPTh): compost and black peat fortified with *T*. *harzianum*; (P_CPTa): compost and black peat fortified with *T*. *asperellum* (P_CP): compost and black peat (P_P): Peat, inoculated with *P*. *nicotianae*.

The suppressive effect of the compost fortified with *T*. *asperellum* (P_CPTa) was significantly (p<0.05) more effective than the one fortified with *T*. *harzianum* (P_CPTa) (Fig 1A). The treatment P_CPTa showed the least percentage of dead pepper seedlings (p<0.05) while, the non-fortified compost and peat (P_CP and P_P) showed the highest dead seedling (Fig 1A).

qPCR data showed significant differences (p<0.05) of ITS copies g^-1^ of *P*. *nicotianae* after 28 days of sowing between treatments (Fig 1B). The lowest number of *P*. *nicotianae* was observed in treatment (P_CPTa) fortified with *T*. *asperellum* (p<0.05), while the treatments with compost treatment (P_CP) showed the highest values (p<0.05) compared with the rest of treatments (Fig 1B).

### Abundance of BCAs in the rhizosphere of different substrates

qPCR showed that the amount of *T*. *asperellum* did not change significantly (p<0.05) with the pathogen after 28 days of inoculation (P_CPTa) (Table 1), while in the absence of the pathogen (*P*. *nicotianae*) (CPTa) the amount of *T*. *asperellum* decreased significantly (p<0.05) (Table 1).

**Table 1:** Quantification of *T*. *asperellum* and *T*. *harzianum* in different treatments at the beginning of the experiment (0 days) and after harvesting (28 days).

			***T*. *asperellum* (Log copies TEF g**^**-1**^**)**		
	**0 days**			**28 days**	
			***P*. *nicotianae***		**Without *P*. *nicotianae***
**CPTh**	nd	**P_CPTh**	Nd	**CPTh**	nd
**CPTa**	4.54±0.07	**P_CPTa**	4.54±0.02	**CPTa**	4.37±0.08
**CP**	nd	**P_CP**	Nd	**CP**	nd
**P**	nd	**P_P**	Nd	**P**	nd
			***T*. *harzianum* (Log copies ITS g**^**-1**^**)**		
	**0 days**			**28 days**	
			***P*. *nicotianae***		**Without *P*. *nicotianae***
**CPTh**	8.70±0.22	**P_CPTh**	8.48±0.23	**CPTh**	8.53±0.13
**CPTa**	nd	**P_CPTa**	Nd	**CPTa**	nd
**CP**	nd	**P_CP**	Nd	**CP**	nd
**P**	4.56±0.24	**P_P**	4.89±0.29	**P**	4.36±0.45

nd: not detected

On the other hand, *T*. *harzianum* did not change with or without pathogen inoculation after 28 days of inoculation (P_CPTh, CPTh) (Table 1). Values were 4 log times higher for *T*. *harzianum* compared with *T*. *asperellum*. Peat (P) also showed natural *T*. *harzianum* but it was significantly lower than the amount of *T*. *harzianum* in compost and did not change along the bioassay with or without pathogen inoculation (P_CPTh, CPTh).

### Bacterial composition of different treatments by NGS analysis

**Fig 2:**
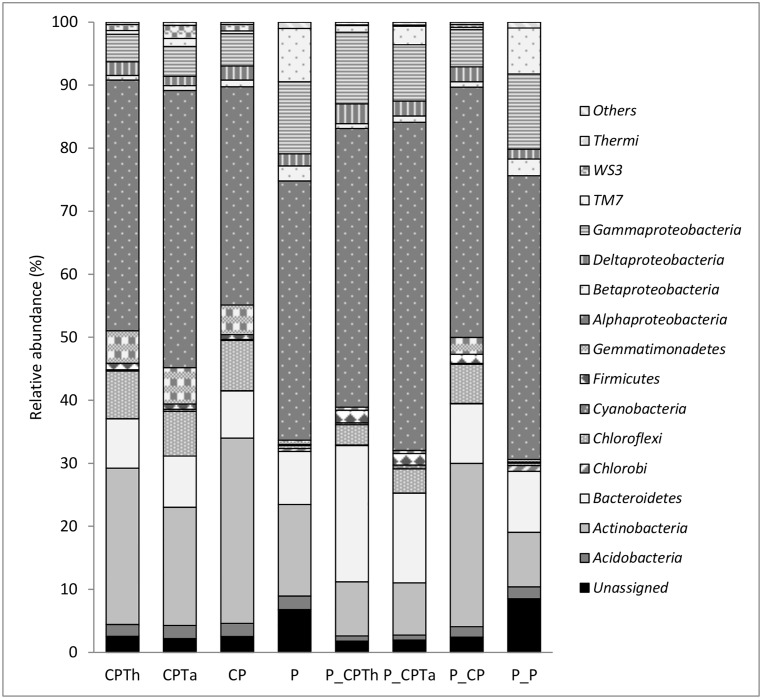
Composition of Bacterial communities at phylum/class level of each treatment after harvesting. (CPTh): compost and black peat fortified with *T*. *harzianum*; (CPTa): compost and black peat fortified with *T*. *asperellum* (CP): compost and black peat (P): peat. Treatments with the letter P_ ahead were the same but inoculated with *P*. *nicotianae*.

After filtering the readings based on quality control, 868.608 sequences with an average length of 181 bases were obtained from 8 samples and assigned to 65.643 OTUS. All of the sequences obtained were classified into 38 phyla and the remaining sequences were classified as unassigned and other bacteria. The relative abundance (>0.5%) for all samples showed *Proteobacteria* as the dominant phylum with *Alphaproteobacteria* (41.93%) and *Gammaprotobacteria* (6.76%) being the most dominant class within that phylum. *Actinobacteria*, *Bacteroidetes* and *Chloroflexi* were also relatively abundant (19.53%, 10.46% and 5.78% respectively) (all accounting of the OTUs in all samples) (Fig 2).

The relative abundance at the phylum level varied across the different samples (Fig 2). Some differences were found between *P*. *nicotianae* non-infected compost treatments (CPTh, CPTa and CP) and peat treatment (P). Peat treatment showed higher relative abundance of *Gammaproteobacteria*, *TM7* and *Chlorobi* and lower relative abundance of *Gemmatimonadetes* and *Chloroflexi*. No differences were shown between fortified composts (CPTh and CPTa) and compost treatment (CP).

Both *P*. *nicotianae* infected fortified composts (P_CPTh and P_CPTa) showed higher relative abundance of *Bacteroidetes* and *Gammaproteobacteria* and lower *Acidobacteria*, *Actinobacteria* and *Chloroflexi* than the non-fortified compost (P_CP). *P*. *nicotianae* infected peat treatment (P_P) showed higher relative abundance of *TM7*, *Chlorobi*, *Acidobacteria* and *Betaproteobacteria*, and lower relative abundance for *Firmicutes* and *Chloroflexi*.

**Fig 3:**
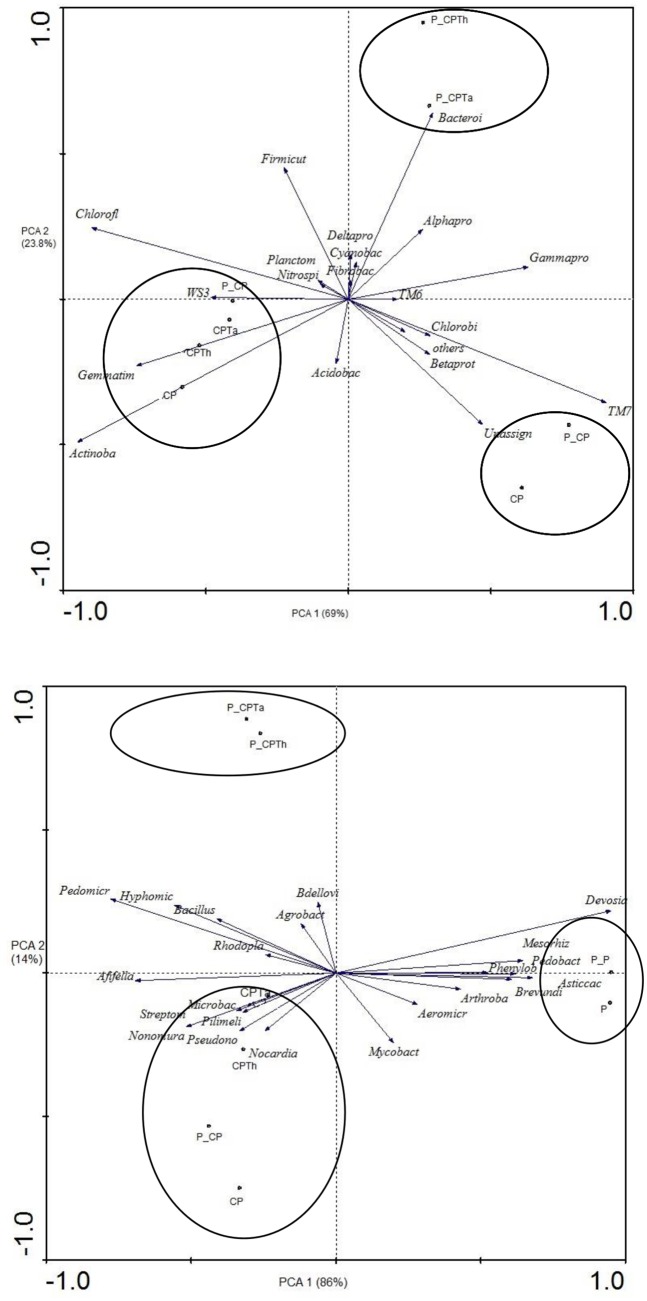
Principal components analysis of bacterial communities (phylum) (A) and genera (B) of all treatments after harvesting. (CPTh): compost and black peat fortified with *T*. *harzianum*; (CPTa): compost and black peat fortified with *T*. *asperellum* (CP): compost and black peat (P): peat. Treatments with the letter P_ ahead were the same but inoculated with *P*. *nicotianae*.

At this level three clear groupings of the samples could be observed on axis 1 (69.2%) of the PCA (Fig 3A), one group that grouped *P*. *nicotianae* infected fortified compost treatment with both *Trichoderma* strains (P_CPTh, P_CPTa); a second group that grouped peat treatments independently of pathogen inoculation (P, P_P); and the third group that grouped the rest of treatments (CP, P_CP, CPTh and CPTa) (Fig 3A).

PCA of the 22 most abundant genera obtained (S2 Table) with a relative abundance (>0.5%) resulted in a similar separation to phylum level on axis 1 (86%) (Fig 3B). A first group of P_CPTh and P_CPTa; a second group of the peat treatments (P and P_P) and the third group containing the other treatments.

### Fungi composition of different treatments by NGS analysis

After filtering the readings based on quality control, 1173993 sequences with an average length of 211 bases were obtained from 8 samples assigned to 216457 OTUS.

Taxonomic assignment of fungal OTUs revealed that at the phylum level, the *Ascomycota* were the most abundant in all treatments (71.39%) followed by *Basidiomycota* (25.28%) and *Zygomycota* (1%) (all accounting of the OTUs in all samples). The percentages of sequences that were classified as unidentified fungi were lower of 2.3% of the OTUs in all samples.

**Fig 4:**
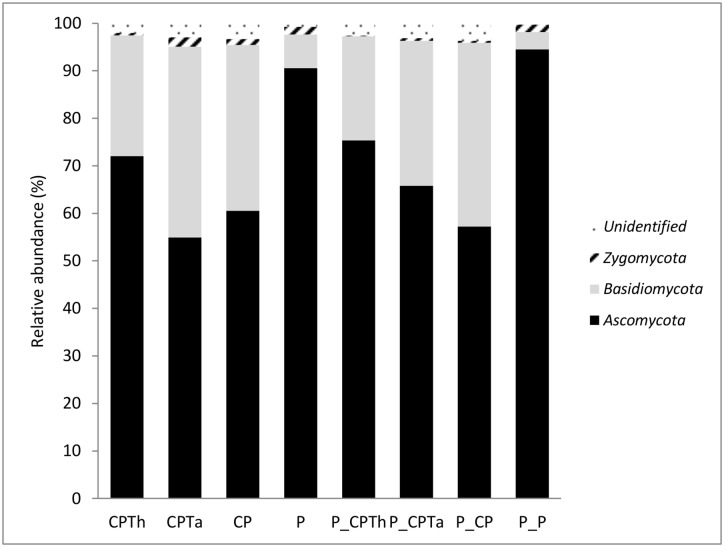
Composition of fungal communities at phylum level of each treatment after harvesting. (CPTh): compost and black peat fortified with *T*. *harzianum*; (CPTa): compost and black peat fortified with *T*. *asperellum* (CP): compost and black peat (P): peat. Treatments with the letter P_ ahead were the same but inoculated with *P*. *nicotianae*.

The relative abundance at the phylum level varied across the different treatments (Fig 4). Independently of *P*. *nicotianae* infection, compost treatments (CPTh, CPTa, CP and P_CPTh, P_CPTa and P_CP) showed higher *Basidiomycota* and lower *Ascomycota* than peat treatments (P and P_P). The relative abundance of *Zygomycota* in the *P*. *nicotianae* infected treatments decreased in all compost treatments (P_CPTh, P_CPTa and P_CP), but not in peat (P_P).

**Fig 5:**
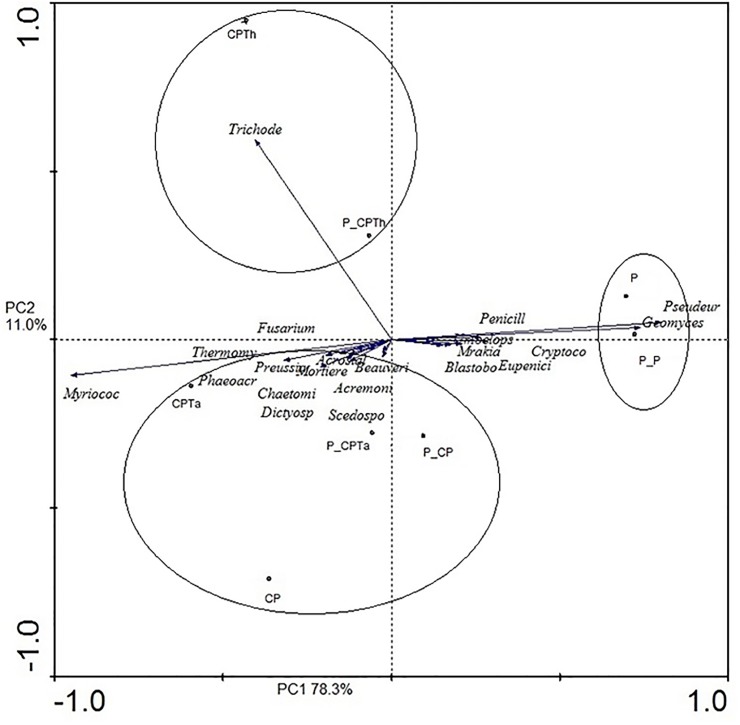
Principal components analysis of fungal communities (genera) of all treatments after harvesting. (CPTh): compost and black peat fortified with *T*. *harzianum*; (CPTa): compost and black peat fortified with *T*. *asperellum* (CP): compost and black peat (P): peat. Treatments with the letter P_ ahead were the same but inoculated with *P*. *nicotianae*.

At the genus level, the PCA of the top 22 classified fungi (>0.5%) (S3 Table revealed that the microbial community varied across the different treatments independently of pathogen. Three groups were observed across Axis 1 (78.3%). One composed of CPTh and P_CPTh; the second formed by the other compost treatments (CPTa, P_CPTa, CP and P_CP); and the third one composed of the peat treatments (P_P and P) (Fig 5).

Sequences reported in this study were deposited in the Genbank (NCBI) databases under accession numbers SAMN 05361899/900/901/902/903/904/905/906.

## Discussion

Compost has been widely used to control plant disease caused by different pathogens such as *Phytophthora* spp. [19], although it is well known that not all composts are suppressive and depend on the extant antagonist microorganisms, plant host, pathogen species involved and the characteristics of compost [28]. The addition of a specific biological control agent to compost has been reported as leading to a substrate with a broader-ranging suppressive effect [29, 30].

Our experiment showed that composts fortified with the two different *Trichoderma* strains showed a lower percentage of dead seedlings, being in both cases significantly more effective against *P*. *nicotianae* than non-fortified compost and peat. The combination of organic substrates and biocontrol agents suppress plant pathogens through different mechanisms which can be summarized as direct interactions such as nutrient and space competence, antibiotic compound production and mycoparasitism; and indirect interaction through plants, such as systemic and acquired resistance (ISR and SAR) [31, 5, 32].

*P*. *nicotianae* abundance measured on *T*. *harzianum* fortified compost did not decreased assuming that no direct pathogen-BCA interaction occurs, and that plant defense induction could be involved [33]. On the other hand, *T*. *asperellum* fortified compost decreased *P*. *nicotianae* abundance and it could be due to a direct pathogen-BCA interaction, probably, producing a mycoparasitism process by *T*. *asperellum* hydrolytic enzyme secretion, causing the hydrolysis of the pathogen cell wall or antibiotic compound production [33]. Furthermore, the population of *T*. *asperellum* fortified compost decreased significantly (p<0.05) after 28 days, while the population of *T*. *harzianum* did not change during the bioassay. This could be due to the different suppressive mechanisms and could be influenced by the composition of the root exudates and the microbes in the rhizosphere where *T*. *harzianum* could be better adapted.

The study of bacteria and fungi communities through 16S and ITS sequencing analysis showed some differences as other authors pointed out [18, 34, 35, 36] which would help to understand the different suppresiveness effects observed.

The bacterial phyla abundance revealed that the most abundant phyla for all treatments were *Alphaproteobacteria* and *Gammaprotobacteria*, documented as typical compost bacteria [37] and *Actinobacteria* and *Bacteroidetes*. Similar results were also observed for different types of composts and peat [34, 35, 36, 38]. Bacterial community from the fortification of compost with the two different *Trichoderma* strains did not show any important differences in the relative abundance of different phyla compared to non-fortified compost. Nevertheless, peat treatments were enriched by *Gammaproteobacterias* [39] and *Candidate division TM7*, as first identified in a German peat bog [40], and as has been shown to be present in soil, sediments, wastewater, animals and a host of clinical environments [41, 42].

PCA of bacterial community showed that the variation at phylum level is more due to the infection with the pathogen than the introduction of *Trichoderma* strains. The interaction between *P*. *nicotianae-Trichoderma* strains and rhizosphere of fortified compost treatment showed *Bacteroidetes* enrichment and in particular the more relative abundance of *Pedomicrobium*, *Hyphomicrobium*, *Bacillus*, *Bdellovibrio* and *Gammaproteobacteria* compared to non-fortified compost treatment, indicating that they may be involved in disease suppression of *P*. *nicotianae*. Similar results were observed by Kyselkova et al. [43] in soil suppressive to tobacco black rot caused by *Thielaviopsis basicola*, or against *R*. *solani* infection especially controlled by *Pseudomonadaceae* [44]. Blaya et al. [38] also demonstrated that these genera were found in pepper rhizosphere when suppressive composts were used against *P*. *nicotianae*, but not in the ones that did not show suppressive effect.

On the other hand, *P*. *nicotianae* infection showed a relative increase of *Actinobacteria* but not only in the fortified compost treatment. This could indicate that in this case these microbial communities have a bigger role in decomposition of organic materials particularly for degradation of macromolecules such as cellulose, hemicellulose, etc. [45] than in a positive impact on plant disease suppression due to a strong ability to produce antibiotic-like compounds [46, 47]. In this sense, Bonanomi et al. [48] concluded that disease suppression was only correlated with *Actinobacteria* in a limited number of experimental cases.

Between the top 22 abundant genera, *Bacillus* was relatively more abundant in compost treatments than in peat. It is characterized as being able to form stable and extensive biofilm [49] composed by secreted antifungal compounds, such as surfactin, bacillomycin and microlactin that protect plants against attack by soil-borne pathogens [49, 50]. Other genera of bacteria that have been found in compost treatment higher than in peat described as biological control were *Streptomyces* [51] and Microbacterium [52].

In the fungal community study, *Ascomycota* and *Basidiomycota* were the most abundant phyla, in agreement with other studies with similar organic materials [53, 38]. *Ascomycota* has been observed during different composting processes [34, 36], where most of the microorganisms were saprophytic and lived on dead organic matter that they help to decompose [54]. The variation at phylum level is due principally to the type of substrate and not to the pathogen infection. *T*. *harzianum* fortified compost showed a different fungal diversity, by increasing *Ascomycota* and by decreasing *Basidiomycota*, to the other two compost treatments.

It can be considered that different genera as *Trichoderma*, *Fusarium* and *Myriococcum* were the most abundant of compost treatment compare to peat independently of pathogen infection that have been related with suppressiveness. *Trichoderma* species and *Fusarium* species has been considered as biological control agent [27, 55] or even species from genera *Myriococcum* has been described to produce antifungal antibiotics [56].

## Conclusion

The use of different biological control agents such as *T*. *harzianum* and *T*. *asperellum* in fortified compost can be more effective to reduce *P*. *nicotianae* symptoms in pepper seedlings than only the compost. In addition, the results showed that *T*. *asperellum* fortified compost turned out to be the most effective combination against the pathogen. Changes in the bacterial rhizosphere community were not attributed to the *Trichoderma* strain but to the pathogen infection. On the other hand, the fungal rhizosphere community depended on the substrate but was not affected by plant infection.

These results suggest that the use of next generation sequencing approaches represents a useful method for studying microbial interactions in the rhizosphere, and it is essential to know the effect of biological control agents such as *Trichoderma* spp. in the plant substrate.

## Supporting information

S1 Table Specificity of primers (TaspFw and TaspRv) and probe (Tasp-Pro) to *T*. *asperellum* by qPCR. (DOC)

S2 Table Most abundant bacterial genera identified (>0.5 relative abundance) of different treatments. (DOC)

S3 Table Most abundant fungal genera identified (>0.5 relative abundance) of different treatments. (DOC)
