# Supplementary material for: Relationship of microbial communities and suppressiveness of Trichoderma fortified composts for pepper seedlings infected by Phytophthora nicotianae
Source: PLoS One. 2017 Mar 27;12(3):e0174069. doi: 10.1371/journal.pone.0174069 (PMC5367787; doi:10.1371/journal.pone.0174069)
Supplement: S1 Table — (DOC) [file pone.0174069.s001.doc]

**S1 Table.** Specificity of primers (TaspFw and TaspRv) and probe (Tasp-Pro) to *T. asperellum* by qPCR.

| **Species** | **Isolate** | **Origin** | **Amplification** |
| --- | --- | --- | --- |
| *T. harzianum* | CECT 20714 (T.78) | UK | - |
| *T. atroviride* | CECT 20513 | Spain | - |
| *T. reesei* | CECT 2414 | Unknown | - |
| *T. saturnisporium* | CECT 20109 | USA | - |
| *T. inhamatum* | CECT 20512 | Spain | - |
| *T. hamatum* | CECT 20103 | India | - |
| *T. longibrachiatum* | CECT 20105 | Egypt | - |
| *T. pseudokoningii* | CECT 2937 | Antartic | - |
| *T. asperellum* | CECT 20539 | Spain | + |
| *T. asperellum* | Unknown | Romania | + |

+ Positive amplification; - Negative amplification
