# Supplementary material for: Relationship of microbial communities and suppressiveness of Trichoderma fortified composts for pepper seedlings infected by Phytophthora nicotianae
Source: PLoS One. 2017 Mar 27;12(3):e0174069. doi: 10.1371/journal.pone.0174069 (PMC5367787; doi:10.1371/journal.pone.0174069)
Supplement: S2 Table — (DOC) [file pone.0174069.s002.doc]

**S2 Table.** Most abundant bacterial genera identified (>0.5 relative abundance) of different treatments

| **Phylum** | **Genus** | **TC1** | **TC2** | **TC3** | **TC4** | **P_TC1** | **P_TC2** | **P_TC3** | **P_TC4** |
| --- | --- | --- | --- | --- | --- | --- | --- | --- | --- |
| *Alphaproteobacteria* | *Rhodoplanes* | 5.82 | 7.36 | 4.72 | 3.80 | 4.11 | 7.11 | 5.34 | 3.99 |
| *Alphaproteobacteria* | *Devosia* | 2.24 | 3.17 | 1.76 | 7.61 | 2.80 | 3.30 | 1.49 | 7.87 |
| *Alphaproteobacteria* | *Pedomicrobium* | 1.36 | 1.25 | 0.97 | 0.02 | 1.83 | 2.46 | 1.75 | 0.01 |
| *Alphaproteobacteria* | *Hyphomicrobium* | 1.08 | 1.13 | 0.88 | 0.12 | 1.68 | 2.09 | 1.53 | 0.11 |
| *Alphaproteobacteria* | *Afifella* | 1.51 | 1.67 | 1.41 | 0.02 | 1.09 | 1.23 | 1.31 | 0.02 |
| *Alphaproteobacteria* | *Mesorhizobium* | 0.30 | 0.31 | 0.22 | 1.87 | 0.37 | 0.39 | 0.28 | 2.02 |
| *Alphaproteobacteria* | *Agrobacterium* | 0.36 | 0.22 | 0.13 | 0.07 | 0.31 | 0.57 | 0.13 | 0.09 |
| *Alphaproteobacteria* | *Asticcacaulis* | 0.00 | 0.00 | 0.00 | 0.73 | 0.00 | 0.00 | 0.00 | 0.88 |
| *Alphaproteobacteria* | *Brevundimonas* | 0.01 | 0.01 | 0.00 | 0.79 | 0.01 | 0.00 | 0.00 | 0.72 |
| *Alphaproteobacteria* | *Phenylobacterium* | 0.02 | 0.01 | 0.01 | 0.59 | 0.02 | 0.02 | 0.01 | 0.67 |
| *Deltaproteobacteria* | *Bdellovibrio* | 0.34 | 0.24 | 0.37 | 0.27 | 1.18 | 0.67 | 0.30 | 0.29 |
| *Fermicutes* | *Bacillus* | 0.41 | 0.26 | 0.38 | 0.01 | 1.00 | 0.65 | 0.49 | 0.00 |
| *Bacteroidetes* | *Pedobacter* | 0.00 | 0.00 | 0.00 | 0.64 | 0.00 | 0.00 | 0.00 | 0.70 |
| *Actinobacteria* | *Streptomyces* | 1.04 | 0.82 | 1.08 | 0.27 | 0.51 | 0.93 | 1.44 | 0.17 |
| *Actinobacteria* | *Pseudonocardia* | 0.70 | 0.58 | 1.00 | 0.15 | 0.39 | 0.40 | 1.10 | 0.06 |
| *Actinobacteria* | *Nonomuraea* | 0.89 | 0.62 | 0.85 | 0.00 | 0.33 | 0.45 | 1.16 | 0.01 |
| *Actinobacteria* | *Mycobacterium* | 0.54 | 0.47 | 0.67 | 0.74 | 0.14 | 0.18 | 0.55 | 0.76 |
| *Actinobacteria* | *Microbacterium* | 0.56 | 0.31 | 0.76 | 0.04 | 0.41 | 0.29 | 0.60 | 0.05 |
| *Actinobacteria* | *Nocardia* | 0.41 | 0.28 | 0.81 | 0.05 | 0.22 | 0.18 | 0.58 | 0.07 |
| *Actinobacteria* | *Aeromicrobium* | 0.17 | 0.32 | 0.15 | 0.72 | 0.03 | 0.08 | 0.11 | 0.29 |
| *Actinobacteria* | *Pilimelia* | 0.33 | 0.25 | 0.36 | 0.00 | 0.14 | 0.15 | 0.54 | 0.01 |
| *Actinobacteria* | *Arthrobacter* | 0.03 | 0.02 | 0.04 | 0.71 | 0.01 | 0.04 | 0.07 | 0.40 |
