# Supplementary material for: Relationship of microbial communities and suppressiveness of Trichoderma fortified composts for pepper seedlings infected by Phytophthora nicotianae
Source: PLoS One. 2017 Mar 27;12(3):e0174069. doi: 10.1371/journal.pone.0174069 (PMC5367787; doi:10.1371/journal.pone.0174069)
Supplement: S3 Table — (DOC) [file pone.0174069.s003.doc]

**S3 Table.** Most abundant fungal genera identified (>0.5 relative abundance) of different treatments

| **Phylum** | **Genus** | **TC1** | **TC2** | **TC3** | **TC4** | **P_TC1** | **P_TC2** | **P_TC3** | **P_TC4** |
| --- | --- | --- | --- | --- | --- | --- | --- | --- | --- |
| *Basidiomycota* | *Myriococcum* | 25.16 | 39.46 | 34.61 | 0.00 | 21.84 | 30.42 | 38.69 | 0.01 |
| *Basidiomycota* | *Cryptococcus* | 0.00 | 0.00 | 0.02 | 5.51 | 0.00 | 0.00 | 0.00 | 2.15 |
| *Basidiomycota* | *Mrakia* | 0.00 | 0.00 | 0.00 | 1.30 | 0.00 | 0.00 | 0.00 | 1.29 |
| *Ascomycota* | *Trichoderma* | 34.64 | 7.15 | 0.35 | 1.03 | 37.19 | 4.18 | 1.16 | 0.23 |
| *Ascomycota* | *Pseudeurotium* | 0.00 | 0.00 | 0.00 | 46.08 | 0.00 | 0.00 | 0.00 | 28.22 |
| *Ascomycota* | *Geomyces* | 0.00 | 0.00 | 0.00 | 26.68 | 0.00 | 0.00 | 0.00 | 32.50 |
| *Ascomycota* | *Phaeoacremonium* | 4.14 | 6.38 | 4.17 | 0.02 | 4.80 | 23.74 | 9.98 | 0.03 |
| *Ascomycota* | *Fusarium* | 5.73 | 6.30 | 9.53 | 0.90 | 7.88 | 4.52 | 2.16 | 8.05 |
| *Ascomycota* | *Dictyosporium* | 1.09 | 3.83 | 2.01 | 0.00 | 2.68 | 6.22 | 4.16 | 0.00 |
| *Ascomycota* | *Preussia* | 1.86 | 2.33 | 2.66 | 0.00 | 0.49 | 0.83 | 1.66 | 0.00 |
| *Ascomycota* | *Scedosporium* | 0.97 | 1.43 | 1.57 | 0.00 | 0.96 | 1.87 | 2.75 | 0.00 |
| *Ascomycota* | *Penicillium* | 0.12 | 0.10 | 0.09 | 5.27 | 0.01 | 0.06 | 0.00 | 3.47 |
| *Ascomycota* | *Thermomyces* | 0.93 | 0.95 | 1.16 | 0.00 | 0.16 | 0.14 | 0.33 | 0.00 |
| *Ascomycota* | *Chaetomium* | 0.94 | 1.16 | 1.68 | 0.00 | 0.74 | 0.51 | 0.08 | 0.00 |
| *Ascomycota* | *Acrostalagmus* | 0.52 | 0.76 | 0.41 | 0.00 | 0.40 | 1.58 | 1.41 | 0.00 |
| *Ascomycota* | *Acremonium* | 0.29 | 0.36 | 0.35 | 0.00 | 0.33 | 1.27 | 0.67 | 0.02 |
| *Ascomycota* | *Beauveria* | 0.18 | 0.29 | 0.44 | 0.00 | 0.29 | 0.74 | 1.16 | 0.00 |
| *Ascomycota* | *Eupenicillium* | 0.00 | 0.00 | 0.00 | 1.63 | 0.01 | 0.03 | 0.00 | 1.24 |
| *Ascomycota* | *Blastobotrys* | 0.00 | 0.00 | 0.00 | 0.69 | 0.00 | 0.00 | 0.00 | 0.32 |
| *Zygomycota* | *Umbelopsis* | 0.00 | 0.00 | 0.00 | 0.79 | 0.00 | 0.00 | 0.00 | 0.74 |
| *Zygomycota* | *Mortierella* | 0.48 | 1.95 | 0.99 | 0.00 | 0.16 | 0.54 | 0.42 | 0.00 |
